# Linking Incomplete Reprogramming to the Improved Pluripotency of Murine Embryonal Carcinoma Cell-Derived Pluripotent Stem Cells

**DOI:** 10.1371/journal.pone.0010320

**Published:** 2010-04-26

**Authors:** Gang Chang, Yi-Liang Miao, Yu Zhang, Sheng Liu, Zhaohui Kou, Junjun Ding, Da-Yuan Chen, Qing-Yuan Sun, Shaorong Gao

**Affiliations:** 1 State Key Laboratory of Reproductive Biology, Institute of Zoology, Chinese Academy of Sciences, Beijing, China; 2 National Institute of Biological Sciences, Beijing, China; 3 Department of Histology and Embryology, Capital Medical University, Beijing, China; University of Barcelona, Spain

## Abstract

Somatic cell nuclear transfer (SCNT) has been proved capable of reprogramming various differentiated somatic cells into pluripotent stem cells. Recently, induced pluripotent stem cells (iPS) have been successfully derived from mouse and human somatic cells by the over-expression of a combination of transcription factors. However, the molecular mechanisms underlying the reprogramming mediated by either the SCNT or iPS approach are poorly understood. Increasing evidence indicates that many tumor pathways play roles in the derivation of iPS cells. Embryonal carcinoma (EC) cells have the characteristics of both stem cells and cancer cells and thus they might be the better candidates for elucidating the details of the reprogramming process. Although previous studies indicate that EC cells cannot be reprogrammed into real pluripotent stem cells, the reasons for this remain unclear. Here, nuclei from mouse EC cells (P19) were transplanted into enucleated oocytes and pluripotent stem cells (P19 NTES cells) were subsequently established. Interestingly, P19 NTES cells prolonged the development of tetraploid aggregated embryos compared to EC cells alone. More importantly, we found that the expression recovery of the imprinted *H19* gene was dependent on the methylation state in the differential methylation region (DMR). The induction of *Nanog* expression, however, was independent of the promoter region DNA methylation state in P19 NTES cells. A whole-genome transcriptome analysis further demonstrated that P19 NTES cells were indeed the intermediates between P19 cells and ES cells and many interesting genes were uncovered that may be responsible for the failed reprogramming of P19 cells. To our knowledge, for the first time, we linked incomplete reprogramming to the improved pluripotency of EC cell-derived pluripotent stem cells. The candidate genes we discovered may be useful not only for understanding the mechanisms of reprogramming, but also for deciphering the transition between tumorigenesis and pluripotency.

## Introduction

Various differentiated somatic cells can be reprogrammed into a totipotent, or at least pluripotent state by somatic cell nuclear transfer (SCNT), which includes fetus-derived epithelial cell lines [1], cumulus cells [2], mature B and T lymphocytes [3], olfactory sensory neurons [4], [5] and natural killer T cells [6]. This reprogramming process requires the reversal of epigenetic modifications, such as DNA methylation, histone modifications, and the condensation state of chromatin [7]. Recently, induced pluripotent stem (iPS) cells were generated by the forced expression of four transcription factors in mouse fibroblasts, and the derived iPS cells are similar to ES cells [8]. However, the detailed mechanisms underlying these complicated reprogramming events are not well understood.

Epigenetic modifications play important roles during the development of embryos and the initiation of disease. The definition of cell fate always coincides with changes in its epigenetic modifications, such as DNA methylation and histone modifications. Aberrant epigenetic modifications could result in many kinds of diseases, such as cancer [9]. For instance, it is well known that the promoter regions of many important tumor suppressor-genes are always hypermethylated, which inactivates the supervisory roles of tumor suppressor genes, thus resulting in the neoplasia [10]. During the reprogramming process mediated by SCNT, genetic alternations cannot be corrected, whereas the epigenetic modifications can indeed be reset.

Embryonal carcinoma (EC) cells, derived from teratocarcinomas, are capable of unlimited self-renewal and can differentiate into several kinds of somatic cells. The isolation of EC cells also provides us with a preliminary framework for embryonic stem cells [11]. Thus, EC cells are widely used as the *in vitro* models for dissecting several fundamental questions related to development and pluripotency [12]. Moreover, the discovery of EC cells demonstrated the existence of the so-called “cancer stem cells” for the first time, predating the current interest in these by several decades. Although EC cells still have similar characteristics to other cancer cells, such as the genetic mutations, they differ from other kinds of cancer cells in the developmental potential, as assessed by the blastocyst injection assay [13].

Previous studies have indicated that some tumor cells are able to direct the development of early cloned embryos, producing morphologically normal blastocysts that give rise to NTES cell lines, but the cloned embryos are not able to develop into live pups after their transfer into the uterus [14], [15], [16]. The failed reprogramming of tumor cells may be due to characteristics of the donor cells, such as the profound genetic changes or the differentiation states of these cells. Accumulated evidence implies that cancer cells may have an inseparable connection with induced pluripotent cells [8], [17], [18]. Considering the special status of EC cells, which have the dual identities of both cancer cells and multipotent cells, we have designed our experiments relying on EC cells. The reprogramming of EC cells may provide an excellent model for understanding the maintenance of tumorigenic potential and pluripotency.

In the present study, we attempted to answer two questions by reprogramming P19 EC cells through SCNT: (I) Can EC cells be reprogrammed and become pluripotent, and to what extent can they be reprogrammed? (II) What molecular events occur during the reprogramming of EC cells?

## Results

### 1. The development of cloned embryos reconstructed with P19 cells and the establishment of P19 cell-derived pluripotent stem cells

**Figure 1:**
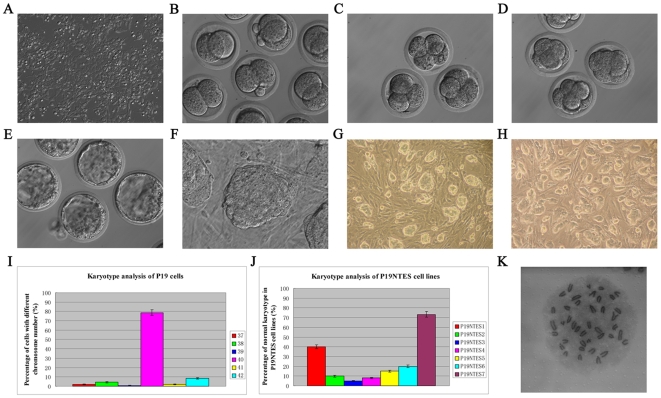
The reprogramming of EC cells and establishment of P19 NTES cell lines. (A) P19 cells cultured *in vitro*. (B) Two-cell stage cloned embryos of P19 cells. (C) Four-cell stage cloned embryos of P19 cells. (D) Eight-cell or morula stage cloned embryos of P19 cells. (E) Reconstructed blastocyst derived from P19 cells. (F) Outgrowth generated from *in vitro* cultured cloned blastocyst of P19 cells. (G) P19 NTES1 cell line at Passage 10. (H) P19 NTES7 cell line at Passage 11. (I) Karyotype analysis of P19 cells. The X-axis represents different chromosome number; the Y-axis represents the percentage of cells with different chromosome number. (J) Karyotype analysis of P19 NTES cell lines. The X-axis represents the serial number of P19 NTES cell line; the Y-axis represents the normal karyotype percentage of each cell line. (K) A typical karyotype of P19 NTES7.

In this study, two tumor cell lines (N2a and P19) with different development potentials were chosen as the donors for the nuclear transplantation. Compared to the N2a cell line, the P19 cell line was highly tumorigenic and in a low differentiation state, characteristics that were verified by the subcutaneous injection into immunodeficient mice (data not shown). P19 cells also always adhered to the culture dish and showed a typical morphology of cancer cells when cultured *in vitro* (Figure 1A).

Nuclei from the two tumor cell lines were injected into enucleated B6D2F1 oocytes and the reconstructed oocytes were activated and cultured to evaluate their *in vitro* developmental capacity. We were unable to obtain cloned blastocysts from 53 reconstructed oocytes containing the nuclei of N2a cells, but we succeeded in producing 171 cloned blastocysts from 628 oocytes after the transfer of nuclei from P19 cells. The development of P19 cell-derived cloned embryos resembled the embryos derived from cumulus cells, which always acted as the control for the routine nuclear transfer assay (Figure 1B, C, D, E). The proportion of cleaved oocytes that reached the blastocyst stage was nearly 30% (Table 1).

**Table 1:** *In vitro* development of embryos after nuclear transfer of N2a cells, P19 cells, and cumulus cells (CCs).

Cell Type	No. of Oocytes	No. of 2-Cell (%)	No. of 4-Cell (%)	No. of Morula (%)	No. of Blastocyst (%)
N2a	53	33 (60.7±12.2)^a^	9 (16.4±6.8)^a^	0 (0)^a^	0 (0)^a^
P19	361	299 (83.6±2.1)^b^	191 (53.4±1.6)^b^	129 (36.1±1.5)^ b^	95 (29.7±1.6)^b^
CCs	452	412 (91.2±2.7)^b^	259 (57.4±6.5)^b^	173 (38.4±11.6)^b^	117 (26.0±5.4)^b^

a, b, c: These values differ significantly from one another (P<0.05).

Because blastocysts reconstructed with P19 cells had a higher developmental rate, we were very interested in whether these cloned blastocysts could maintain the full-term development of embryos. Therefore, cloned blastocysts derived from P19 cells were transferred back into the uteri of pseudo-pregnant mice, but we failed to get full-term pups. Then we tried to obtain cloned pups through the “two-step” cloning method, which was efficient in overcoming the underlying handicaps that resulted from the direct cloning [3], [4]. First, seven NTES cell lines were established from 21 cloned blastocysts derived from P19 cells with a rate of about 33.3%, which was comparable to the rate of cell lines derived from fertilized embryos (data not shown). These NTES cells from the P19 cells all had the typical morphology of normal ES cells and could be passaged *in vitro* long-term (Figure 1F, G, H). As tumor cells always had karyotype problems, we eliminated the impact resulting from abnormal karyotypes by selecting normal ES cell lines. As seen from Figure 1I, the karyotypes of the P19 cells were relatively normal, 78.7% of which contained 40 chromosomes. Among the P19 NTES cells, however, only P19 NTES7 cells showed relatively normal karyotypes (73.3%) and this line was thus selected for subsequent experiments (Figure 1J, K).

### 2. Pluripotent P19 NTES cells dramatically prolong the development of tetraploid aggregated embryos

**Figure 2:**
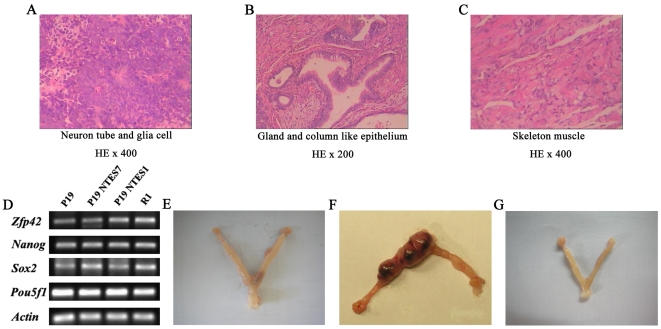
Pluripotency verifications of P19 NTES cells. Teratoma formation, RT-PCR and tetraploid aggregated assays were used to evaluate the pluripotency of P19 NTES cells from different aspects. (A), (B), (C) HE staining results of the paraffin embedded sections of teratoma derived from P19 NTES7. As shown in A-C, neuron tube and glia cells, gland, and column-like epithelium and skeleton muscle, which represent the three germ layers, respectively, could be observed in teratoma tissues. (D) The expression of pluripotent genes of P19 and P19 NTES cells as determined by RT-PCR. (E) The uterus and the dead tetraploid embryos of P19 cells at E8.5. (F) The uterus and the dead tetraploid embryos of P19 NTES7 at E8.5. (G) The uterus transplanted with tetraploid embryos derived from P19 NTES1 at E8.5.

P19 NTES cells maintained the typical morphology of normal murine ES cells when cultured *in vitro* long-term. After this period of culturing, we performed teratoma formation and tetraploid blastocyst aggregation assays to verify their pluripotency. After being subcutaneously injected into SCID mice for three to four weeks, P19 NTES cells could form teratomas with a high frequency. The histology of the resulting tumors from P19 NTES cells exhibited differentiation into neural and glial cells (Figure 2A), gland and column-like epithelia (Figure 2B), and skeletal muscle (Figure 2C). Moreover, the expression of pluripotent genes, such as *Zfp42*, *Nanog*, *Sox2* and *Pou5f1,* were also observed in P19 NTES cells, although P19 cells also had a high background expression of these pluripotent markers (Figure 2D).

The EC cells joined in the formation of somatic tissues after they were injected into blastocysts [13]. Therefore, the detection of three germ layers in the teratoma tissues could just reflect the *in vivo* differentiation potential of P19 NTES cells, not their real pluripotency. It was also unknown whether pluripotent stem cells derived from the EC cells could eliminate their tumorigenic potential and maintain the full-term development of the tetraploid chimera, which was the stringent standard for pluripotency. Therefore, tetraploid blastocyst aggregation assays were performed with P19 cells and P19 NTES cells separately. Among the 121 blastocysts injected with P19 cells, only four placentas under absorption were observed at embryonic day (E) 8.5, which was consistent with the results of previous studies, and no live fetuses could be isolated from the absorbing tissues after C-section (Figure 2E and Table 2). In contrast to P19 cells, P19 NTES7 could prolong the development of tetraploid aggregated embryos dramatically. As shown in Figure 2F and Table 2, tetraploid aggregated embryos derived from P19 NTES7 always had bigger placentas than those of P19 cells and the implantation rate was very high, which was a direct indication of increased pluripotency. Although most of these implanted tetraploid complementary embryos derived from P19 NTES7 died before E8.5, we were able to obtain six live E8.5 fetuses of P19 NTES7 from the pseudo-pregnant mice via C-section (Table 2). In contrast to P19 NTES7 cells, P19 NTES1 cells failed to maintain the early development of tetraploid aggregated embryos beyond E8.5 (Figure 2G and Table 2). Therefore, although P19 NTES7 also failed to maintain the full-term development of tetraploid aggregated embryos, it did have an increased pluripotency compared to its progenitor P19 cells.

**Table 2:** The development of tetraploid mice derived from P19, P19NTES7 and P19NTES1 cells.

Cell type	Injected blastocyst	Implantation site	Embryos Arrested before E8.5	Live pups at E8.5	Full term pups
P19	121	4	4	0	0
P19 NTES7	459	231	225	6	0
P19 NTES1	156	0	0	0	0

These data indicated that P19 NTES cells had an increased pluripotency compared to their progenitor cells and the improved pluripotency could extend the development of tetraploid aggregated embryos dramatically. However, not every P19 NTES cell line had the similar improved pluripotency of P19 NTES7, with P19 NTES1 as the typical example.

### 3. The results of molecular analyses indicate that P19 NTES cells are intermediate between EC cells and ES cells

**Figure 3:**
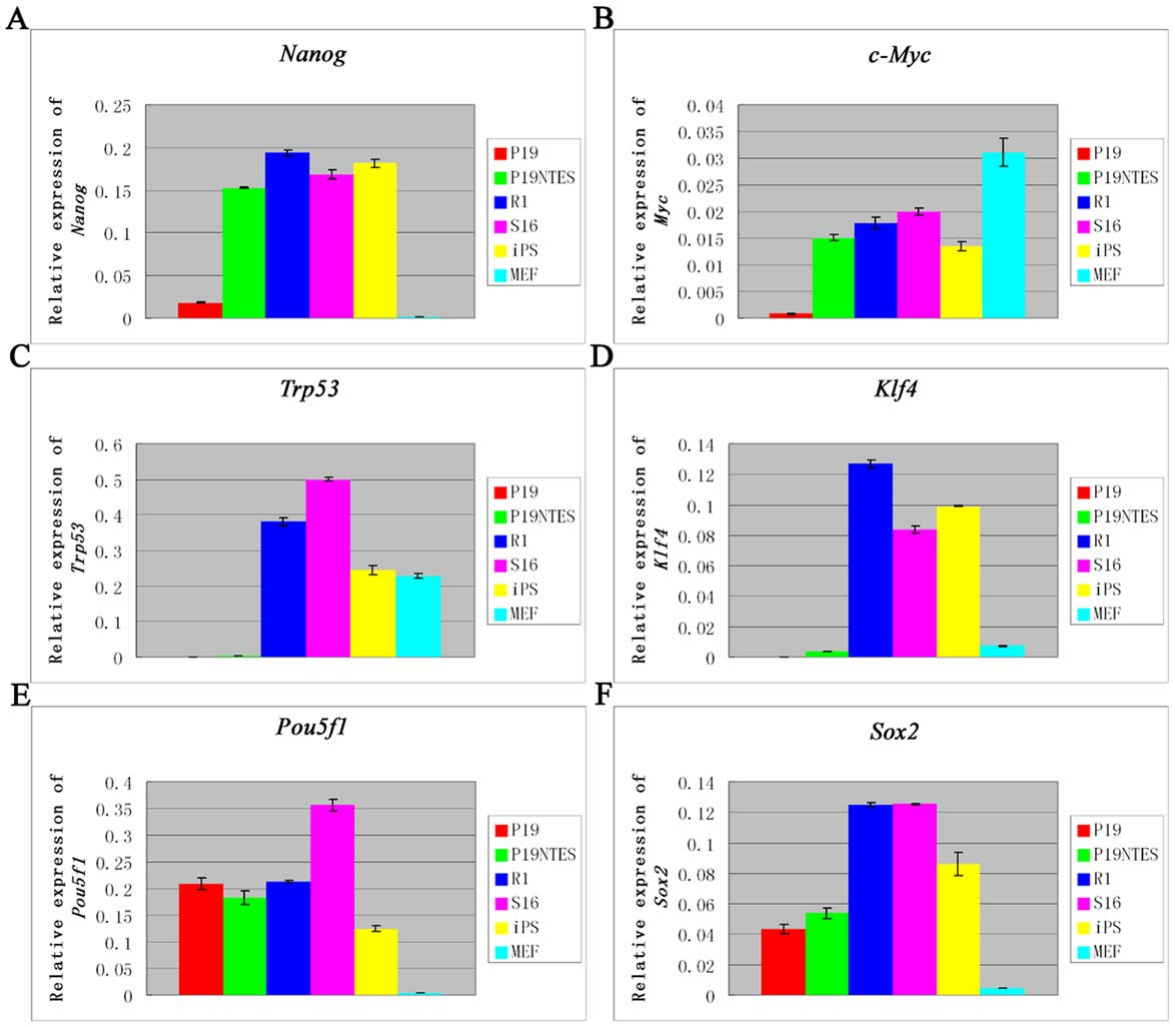
The comparison of P19 NTES cells to normal ES cells, NTES cells, iPS cells and MEF. Quantitative PCR was used to assess the difference among these cells at the level of mRNA by analyzing several important genes. (A), (B) *Nanog* and *c-Myc* are representative of genes that could be induced in P19 NTES cells successfully. (C), (D) *Trp53* and *Klf4* are representative of genes that could not be induced in P19 NTES cells. (E), (F) *Pou5f1* and *Sox2* are genes that did not change during the process of EC reprogramming. Error bars depict standard deviation. p-value<0.01.

Because P19 NTES cells showed an improved pluripotency as compared with progenitor cells, we hypothesized that undiscovered molecular events lead to this cell fate transition. We thus decided to characterize this phenomenon by thoroughly analyzing the molecular characteristics of P19 NTES cells. We used real-time PCR and microarrays to generate a global view of the transcriptional change during the reprogramming process of P19 cells. First, we analyzed the expression of many cancer or pluripotency-related genes in P19 NTES7 cells with several specific cell lines as controls. These cell lines included the normal ES cells (R1), which are the gold standard of true pluripotency, NTES cells derived from Sertoli cells (S16), iPS cells generated from fibroblasts (iPS) and mouse embryonic fibroblasts (MEF). As shown in Figure 3A-F, we classified these genes into three groups. The first group included the genes that could be induced during the reprogramming process of P19 cells by NT, with *Nanog* as the representative gene. The second group comprised the genes that could not be induced to the expression level of normal ES cells, with *Trp53* as the example. The third group contained the genes with no expression change during this process, such as *Pou5f1* and *Sox2.* Thus, we had direct evidence supporting our hypothesis that P19 NTES7 had a unique molecular code, which was worthy of a deeper analysis.

**Figure 4:**
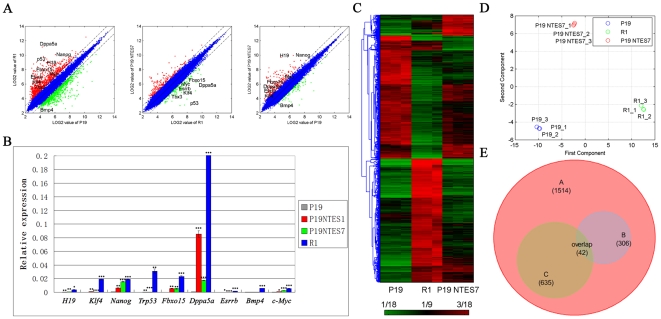
Molecular characteristics of P19 NTES cells. Scatter plot analysis was performed to compare the global gene expression profiles among different groups; hierarchical clustering and overlapping were further carried out to sort and analyze the differentially expressed genes. (A) Scatter plot analysis of the differentially expressed probe sets among P19 cells, P19 NTES cells and R1. Blue dots represent the unchanged probe sets; red dots represent the up-regulated probe sets; green dots represent the down-regulated probe sets. (B) Verification of the expression of several important genes undergoing dramatic changes by quantitative PCR. Error bars depict standard deviation. * p-value <0.05; ** p-value <0.01; *** p-value <0.001 (C) Unsupervised hierarchical clustering of averaged global transcriptional profiles obtained from P19 cells, P19 NTES cells and R1. (D) PCA. (E) Overlapping analysis of GeneSets A, B, and C.

To test this hypothesis, we used microarray for analyzing the transcriptome of P19 NTES cells to help us understand the improved pluripotency detected (Accession number: GSE18691). The microarray results were first displayed as scatter plots, in which the differentially expressed probe sets were marked by red and green dots, while the unchanged probe sets were marked by blue dots. As shown in Figure 4A, P19 cells had a distinct expression pattern compared to R1 ES cells, and these differentially expressed probe sets represented the difference between EC cells and ES cells. In contrast to the distinct expression pattern in the P19 *versus* R1 group, the P19 NTES7 *versus* P19 group and the P19 NTES7 *versus* R1 group had fewer differentially expressed probe sets. In order to reduce the noise in our analysis, we increased the threshold level (fold change>2, one-way analysis of variance (ANOVA) p-value<0.01). Among the probe sets covered in the GeneChip® Gene 1.0 ST Array System for the mouse genome, we found 1641 differentially expressed probe sets between P19 cells and R1 representing 1514 genes (Dataset S1). The sorted genes included several important genes, such as *BMP4*, *Fbxo15*, *Dppa5a*, *Esrrb*, *Trp53*, *Nanog*, *Klf4*, *H19*, *Tbx3* and *c-Myc*. Nearly all of these genes met the more stringent selection standard of significance (fold change>5, one-way ANOVA p-value<0.001), with *c-Myc* as an exception for its special role in both tumorigenesis and reprogramming. Interestingly, these genes always related to pluripotency (*BMP4*, *Fbxo15*, *Dppa5a*, *Nanog*) and tumorigenesis (*Trp53*, *Klf4*, *H19*, *c-Myc*). The expression patterns of these genes were verified by real-time PCR with P19 cells as the reference. As shown in Figure 4B and Dataset S2, the results of the quantitative PCR corresponded well to the microarray results (Max fold change>2.5, p-value<0.01) (Table 3). Among these genes, *Nanog* and *H19* in P19 NTES7 were the outstanding ones because they were successfully induced to the normal expression level, while *Fbxo15*, *Dppa5a*, *Esrrb*, *Klf4* and *c-Myc* were only induced to an intermediate level. In striking contrast, *BMP4*, *Trp53* and *Tbx3* still maintained the expression patterns of EC cells, without any induction trend. As we know, these genes are all involved in critical signaling pathways, and the dysfunction of any of these genes will lead to tumorigeneis. Altogether, these results suggested that P19 NTES7 cells were really the intermediate between P19 cells and R1 cells and this intermediate had the identities of partial pluripotent stem cells, not only at the level of development potential, but also at the molecular level. Taking this into consideration, we decided to further explore the hierarchy among them.

**Table 3:** Transcriptional profiles of specific genes in the Microarray Database.

Gene	P19 mean	P19NTES7 mean	R1 mean	Fold Change (max)	P-value	Gene Description
*BMP4*	14.2228	12.7877	113.1357	8.847228	3.77E-07	Bone morphogenetic protein 4
*Fbxo15*	13.4023	66.3999	304.1208	22.691687	1.13E-06	F-box protein 15
*Dppa5a*	19.5185	125.3853	2238.42	114.681958	5.33E-07	Developmental pluripotency associated 5A
*Esrrb*	9.2619	32.4116	229.8209	24.813580	1.96E-08	Estrogen related receptor, beta
*P53*	11.393	11.1797	531.9999	47.586241	7.53E-12	Transformation related protein 53
*Nanog*	177.6313	626.9135	913.3968	5.142094	8.23E-07	Nanog homeobox
*Klf4*	13.0388	41.4887	125.5365	9.627918	5.58E-08	Kruppel-like factor 4 (gut)
*H19*	14.8403	172.4851	108.9784	11.622750	2.11E-05	H19 fetal liver mRNA
*Tbx3*	12.4563	19.7289	65.3785	5.248629	3.47E-05	T-box 3
*c-Myc*	61.274	86.9802	157.5109	2.570599	0.0029226	Myelocytomatosis oncogene

Next, an unsupervised hierarchical clustering analysis was performed to assess the similarities in single gene expression profiles in an unbiased way among the P19, P19 NTES7 and R1 cells and all the differentially expressed genes were covered (Figure 4C). In the resulting sample tree, P19 cells and R1 cells separated very well and there was nearly no clustering between them. This may account for the intrinsic difference between cancer cells and ES cells. However, P19 NTES7 showed a different schema when compared to either P19 cells or R1. In the top and bottom of the resulting sample tree, P19 NTES7 clustered with R1 cells, while in the middle region, P19 NTES7 clustered with P19 cells. Hierarchical clustering revealed the intermediate state of P19 NTES7 at the gene expression level.

A principal components analysis (PCA), an exploratory multivariate statistical technique always used to simplify complex data sets, was used to evaluate the microarray data. Using this approach, similar samples should be close in distance [19]. As seen from Figure 4D, the three replicates of each cell line were all the closest in the PCA and the separation among the three kinds of cell lines was clear. Therefore, the PCA analysis indicated that the repeatability of microarray data was reliable and the main variable component came from the origin of every cell line.

In summary, these experiments demonstrated that P19 NTES cells had unique molecular characteristics and they were indeed the intermediates between EC cells and ES cells. The gain of function of many pivotal genes in P19 NTES cells coming from partial reprogramming might account for the extended development of tetraploid aggregated embryos observed in the earlier experiments.

### 4. Gene Ontology and pathways enrichment of differentially expressed genes among P19 cells, P19 NTES cells and normal ES cells

In order to understand functions of differentially expressed genes among the P19, P19 NTES7 and R1 cells, we pursued an alternative comparative approach and a gene ontology (GO) analysis was used to identify categories within which genes differed significantly in these cells. First, we screened the differentially expressed genes from P19 *versus* R1 and this set of genes was named as GeneSet A or A (Dataset S1) (fold change >2, one-way ANOVA p-value <0.01). Then we further sub-classified GeneSet A into two sub-groups. The first was named as GeneSet B or B (Dataset S3), and genes in this set met the significance standard for GeneSet A and the one for P19 *versus* P19 NTES7 (fold change >2, one-way ANOVA p-value <0.01). Genes in GeneSet C or C (Dataset S4) met the significance standard for GeneSet A and the one for P19 NTES7 *versus* R1 (fold change >2, one-way ANOVA p-value <0.01). Under this stringent classification standard, GeneSet A was regarded as the molecular differences between EC cells and normal ES cells, GeneSet B represented the genes that were successfully reprogrammed during EC cells reprogramming, and GeneSet C represented the genes which have not been successfully reprogrammed.

The reliability of this classification was confirmed by the overlapping results in Figure 4E and Dataset S5. Among the 1514 genes included in GeneSet A, GeneSet B contained 306 genes and GeneSet C contained 635 genes. GeneSet B and GeneSet C were also well separated with only 42 overlapping genes. Therefore, this sub-classification standard could provide us with more reliable information for further analyzing the functions of these genes.

**Figure 5:**
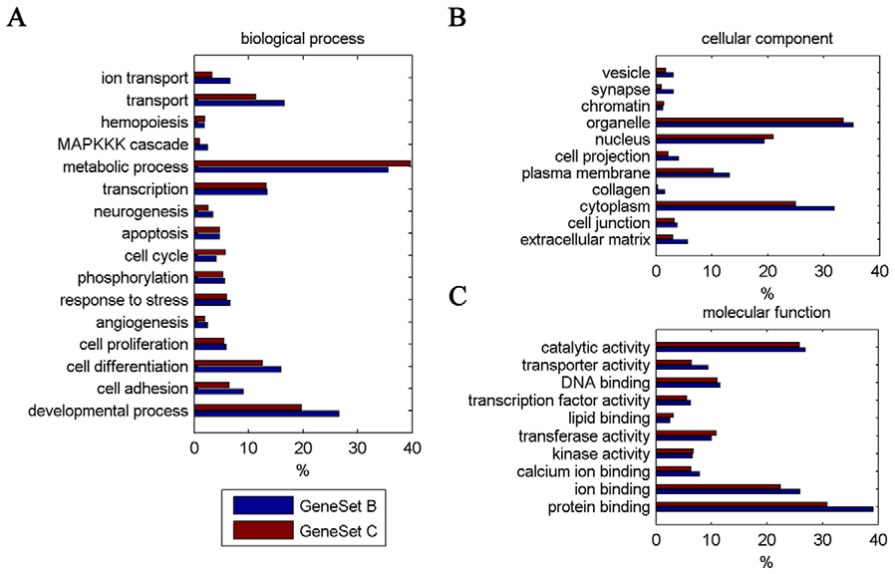
Functional annotation of the differentially expressed genes during the incomplete reprogramming of P19 cells. Gene Oncology analysis was performed to identify functional categories overrepresented in the cluster of genes of GeneSet B and C. (A) Gene Oncology analysis of Group B and Group C according to biological process. (B) Gene Oncology analysis of Group B and Group C according to cell component. (C) Gene Oncology analysis of Group B and Group C according to molecular function. The blue column represents Group B; the red column represents Group C. The X-axis represents the percentage of each sub-category in this category. The Y-axis represents a detailed description of the roles of each sub-category. p-value<0.05.

Next, GO analysis was used to identify the functional categories overrepresented in the cluster genes from GeneSet B and GeneSet C. As shown in Figure 5A-C, the functional categories identified from the two groups of genes were very similar and the enriched categories included several biological processes, such as the developmental process, metabolic process, transcription, transport and cell differentiation. Intriguingly, these biological processes corresponded to several molecular functions, for instance, protein binding, catalytic activity and ion binding. When we reduced the scope by comparing the functional categories covered in GeneSet B and C, we found that the protein binding activity was the only different one (p-value <0.05). The full list of GO analyses can be found in the supporting information, Dataset S6, Dataset S7, and Dataset S8. Overall, our data indicated that GeneSet B and GeneSet C converged on similar functional categories and the enriched activities may be prevalent in the transition of EC cells into ES cells.

A pathways enrichment analysis was then performed to refine the differentially expressed genes into specific signaling pathways during the reprogramming process. As shown in Dataset S9, GeneSet A covered six conventional signaling pathways, including the MAPK, focal adhesion, P53, ECM-receptor interaction, tight junction and VEGF signaling pathways, and these pathways needed to be recovered when the EC cells were reprogrammed into pluripotent stem cells (p-value <0.05). Regarding GeneSet B and C, we found the pathways covered in GeneSet B were reduced dramatically and only contained the MAPK, ECM-receptor interaction and focal adhesion signaling pathways. Nevertheless, GeneSet C could map to the tight junction, focal adhesion, VEGF and p53 signaling pathways, which was in contrast to GeneSet B (Dataset S10, Dataset S11) (p-value <0.05). The recovered genes mainly fell into the MAPK, ECM-receptor interaction and focal adhesion signaling pathways. For instance, *c-Myc*, the downstream member of the MAPK pathway, was induced partially while the focal adhesion signal pathway could only be repaired partly. Most importance of all, the P53 pathway failed to be recovered, with many critical genes inactive, such as *Trp53*, *Gadd45* and *Cdkn1a* (Figure S1, Figure S2). The role of P53 was complex for its non-protecting function in germ cell tumors and negative expression in germ cells and sertoli cells [20], [21]. Therefore, P19-derived pluripotent stem cells still bear the similar attributes of cancer cells and a P53 independent mechanism may exist for protection. In brief, the pathways enrichment results suggested that the reprogramming of EC cells through NT could repair the MAPK, ECM-receptor interaction and focal adhesion signaling pathways, at least partially, but not the P53 pathway.

### 5. The expression recovery of *H19* is dependent on methylation in the differential methylation region (DMR), while the induction of *Nanog* is independent of the promoter region methylation

As showed in the data thus far, P19 NTES cells resulted from an incomplete reprogramming, with many genes fully induced, such as *H19* and *Nanog*, and many genes partially induced, such as *Fbxo15*, *Dppa5a* and *Esrrb*. However, the mechanisms underlying the induction of these genes were unclear, especially for the representatives of the fully induced genes, *H19* and *Nanog*. Combined with the GO results, which indicated that GeneSet B always took part in the protein binding, we reasoned that specific epigenetic transformations may have been involved in the reprogramming of the EC cells.

**Figure 6:**
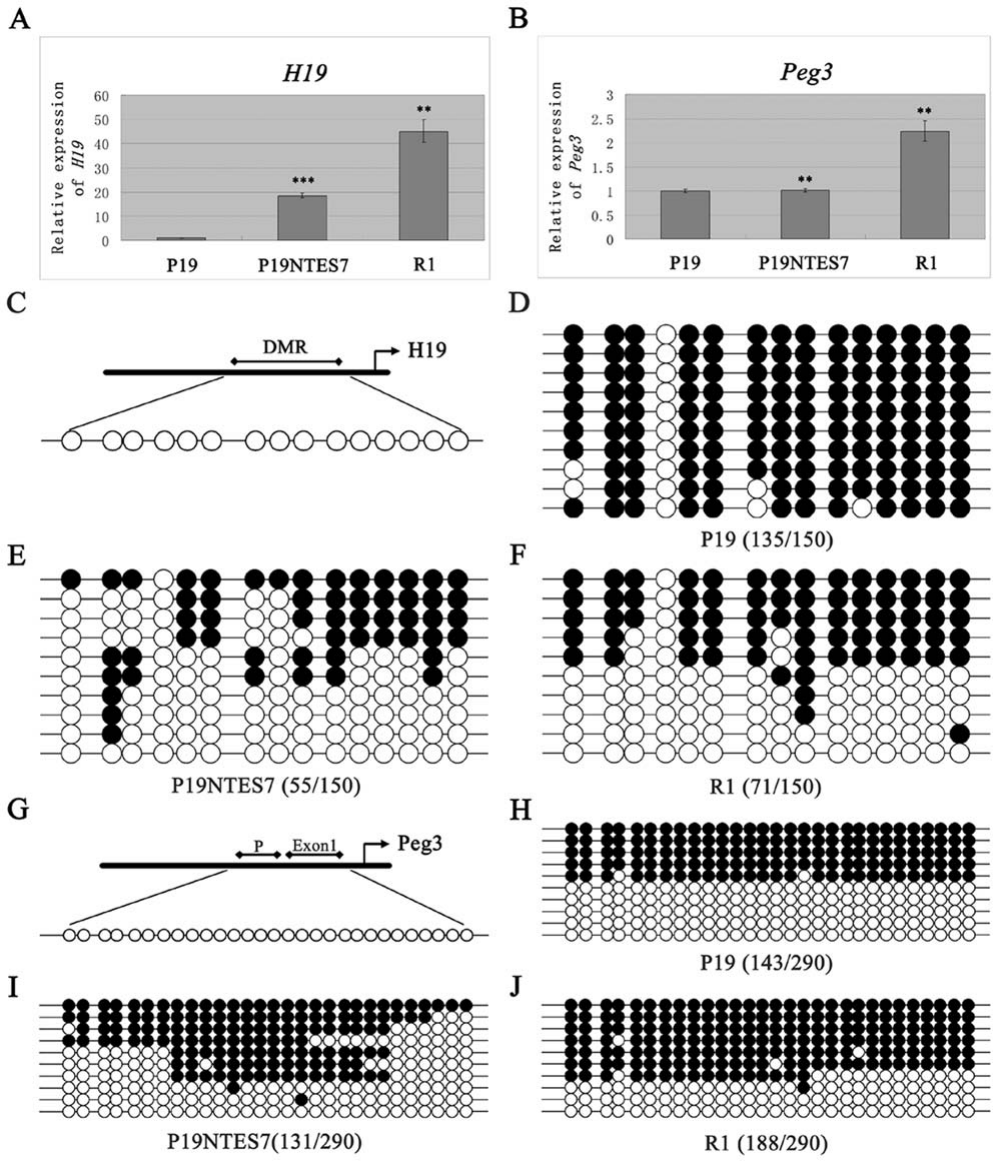
The expression analysis of *H19*, *Peg3* and DNA methylation analysis of their specific DMRs. In order to understand the relationship between the induction of *H19* and DNA methylation of its DMR, bisulfite genomic sequencing was performed among P19, P19 NTES7 and R1, with *Peg3* as control. (A), (B) The expression analysis of *H19* and *Peg3* determined by quantitative PCR. Error bars depict standard deviation. ** p-value<0.01; *** p-value<0.001 (C) The CpG islands contained in DMR of *H19* are shown schematically. (D), (E), (F) The bisulfite genomic sequencing results of *H19* in P19, P19 NTES7 and R1 are shown. (G) The CpG islands contained in DMR of *Peg3* are shown schematically. (H), (I), (J) The bisulfite genomic sequencing results of *Peg3* in P19, P19 NTES7 and R1 are shown schematically. The black circle represents the methylated CpG island, the white circle represents the unmethylated CpG island.

In order to test this hypothesis, we analyzed the DNA methylation changes during the induction of *H19* and *Nanog* using the bisulfite genomic sequencing method. Moreover, western blot was also performed to analyze the expression status of *Nanog* during the process of reprogramming, but not *H19* due to its protein non-coding property [22]. As shown in Table 3 and Figure 6A, the expression of *H19* could be recovered to a normal level. The expression of the other imprinted gene, *Peg3*, had no change, which has been verified by the microarray and real-time PCR (Figure 6B). Next, the DMRs of *H19* and *Peg3* were analyzed for their DNA methylation patterns. Interestingly, the methylation in the DMR of *H19* in P19 cells was very high (135/150), while the levels in P19 NTES7 and R1 were within the normal range (55/150, 71/135) (Figure 6C, D, E, F). In contrast to *H19*, there was no change of the DNA methylation profiles of *Peg3* among these cell lines and it acted as a better control for *H19* (Figure 6G, H, I, J). The observed hypermethylation of P19 cells corresponded to the repression of *H19* and the induction of *H19* was dependent on the methylation in the DMR.

**Figure 7:**
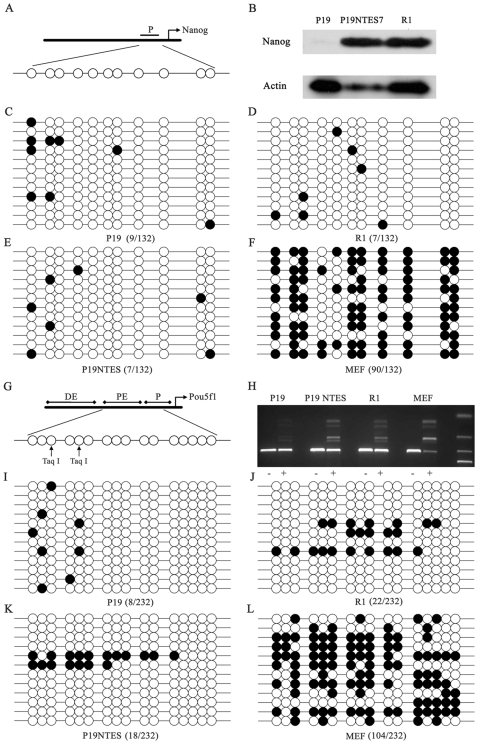
The expression analysis of *Nanog*, *Pou5f1* and DNA methylation analysis of their specific promoter regions. In order to investigate the induction mechanisms of *Nanog*, bisulfite genomic sequencing was performed to analyze DNA methylation changes of its promoter region, which is the well-known regulatory element of *Nanog*, among P19, P19 NTES7 and R1, with *Pou5f1* as control. (A) The CpG islands in the promoter region of *Nanog* are shown schematically. (B) The expression analysis of Nanog in P19, P19 NTES7, R1 and P19 NTES1 determined by western blot. From left to right, P19, P19 NTES7, R1 and P19 NTES1 are listed sequentially. (C), (D), (E), (F) The bisulfite genomic sequencing results of *Nanog* in P19, P19 NTES7, R1 and MEF are shown schematically. (G) The CpG islands in the promoter region of *Pou5f1* are shown schematically; and the arrows show the recognition sites of Taq I. (H) The gel electrophoresis results of *Pou5f1* digested by Taq I. (+): digested and (−): undigested. From left to right, P19, P19 NTES7, R1 and MEF are listed sequentially. The black circle represents the methylated CpG island; the white circle represents the unmethylated CpG island.

Similar to *H19*, the mRNA level of *Nanog* was also induced to a relatively normal level in P19 NTES cells (Table 3 and Figure 3A). Western blot results further confirmed the microarray and real-time PCR data (Figure 7B). We also analyzed the DNA methylation profiles in the promoter region of *Nanog*, with *Pou5f1* as the control (Figure 7A, G). As shown in Figure 7C, D, E, F, the methylation of *Nanog* in P19, P19 NTES7 and R1 were all very low (9/132; 7/132; 7/132) while the methylation level in MEF was very high (90/132). *Nanog* was one of the important factors in the generation of iPS cells and it also played major roles in maintaining pluripotency. The regulation of *Nanog* always relied on the methylation state in the promoter region, with hypomethylation resulting in activation and hypermethylation resulting in silence. Therefore, our results provided direct evidence for the existence of a specific regulatory circuit in controlling the expression of *Nanog* in EC cells and the recovery of *Nanog* was the remarkable indicator of the improved pluripotency in P19 NTES cells. When we analyzed the methylation patterns of *Pou5f1*, we found that the unchanged expression of *Pou5f1* correlated with the ubiquitous hypomethylation of the promoter region in P19, P19 NTES7 and R1, with MEF as the negative control (Table 3 and Figure 7I, G, K, L). The DNA sequencing results were further confirmed by restriction enzyme digestion assays (Figure 7H).

In summary, the induction of *H19* during the partial reprogramming of EC cells was in a DMR methylation-dependent pattern while the induction of *Nanog* was independent of the promoter region methylation.

## Discussion

In this study, we addressed two questions that remain regarding the reprogramming of embryonal carcinoma cells by using a “two-step” cloning approach. Our results demonstrate that P19 NTES cells have an improved pluripotency compared to their progenitor cells, as we observed in the tetraploid aggregated embryos assays. The gain of function of several pluripotent genes identified by high-throughput methods may account for this phenomenon. However, the reprogramming of EC cells is indeed incomplete and the partial reprogramming of P19 cells mainly results from the characteristics of the donor nuclei, such as the specific epigenetic modification patterns, with no direct relationship to the reprogramming process itself. Strikingly, the induction of important genes in EC cells correlates with different regulatory mechanisms, with *H19* in a DMR methylation-dependent pattern and *Nanog* in a promoter region methylation-independent pattern. Therefore, the successful reprogramming of EC cells is still challenging and further investigation of the regulatory circuits involved in the reprogramming of EC cells will help us to ultimately conquer this fundamental question.

Previous attempts at reprogramming tumor cells proved that it was difficult to reprogram tumor cells or get stable ES cell lines from cloned blastocysts [14], [15], [16]. Therefore, tumor cells might have different attributes that result in the failed reprogramming. It was reported that EC cells carry many genetic mutations and these unrecoverable changes may partially explain the failure in the cloning of EC cells [16]. EC cells have a limited pluripotency, and they may be easy to reprogram, as suggested by the accumulating evidence from ES cell cloning and the derivation of iPS cells [23], [24]. We observed that early embryos derived from P19 cells had a similar developmental rate to those generated from cumulus cells and ES cell lines could easily be derived from the cloned blastocysts. Interestingly, one of the derived P19 NTES cell lines (P19 NTES7) had an improved pluripotency and it could prolong the development of tetraploid aggregated embryos. We succeeded in obtaining live E8.5 tetraploid aggregated embryos, which was our strongest evidence for the improved pluripotency. Compared to the previous report [16], we employed here tetraploid complementation assay to evaluate the improved pluripotency of P19 NTES cells because the previous study used chimera analysis through which they could not detect obvious improved pluripotency after NT of P19 cells. The improved pluripotency we observed was verified by the microarray analysis, by which many biomarkers during this transition process were detected. Although the improved pluripotency was not sufficient, it was the basis for the final success in transforming tumor cells into real pluripotent stem cells.

Recently, new types of pluripotent cells, such as iPS cells and germline-derived pluripotent stem (gPS) cells have been derived under specific induction conditions [8], [25], [26], [27], [28], [29], [30]. The emergence of so many pluripotent cells provided us with models not only for investigating the occurrence of many regenerative diseases, but also for investigating the reprogramming mechanisms. Compared to the derivation of iPS and other pluripotent cells, the “two-step” cloning that relies on the cytoplasm of the MII oocyte and further *in vitro* induction could effectively reprogram cells in a short time. Currently, the mechanism of reprogramming is still a mystery and we prefer the hypothesis proposed by Yamanaka [31]. Of note, based on previous work [17], he hypothesized that the transformation resulting from *c-Myc* and *Klf4* was followed by a pluripotent cell induction process regulated by *Pou5f1* and *Sox2*. Recently, the role of *Trp53* in the generation of iPS cells strengthened this hypothesis and the emergence of cancer-like cells that act as an intermediate may be indispensable for getting pluripotent iPS cells successfully [18], [32], [33], [34], [35]. Similarly, we found that *Trp53* and the members of P53 signaling pathways were still inactive in EC cell-derived pluripotent stem cells. In a sense, the repression of the P53 signaling pathway further indicated that EC-derived pluripotent stem cells had different identities from real pluripotent stem cells. Meanwhile, the incomplete or failed induction of *c-Myc* and *Klf4* were also observed, which may due to the inactivity of *Trp53*. EC cells always had high levels of wildtype P53, which was in a repressed state modulating by an N-terminal repression domain; and the repression state would be activated under the induction of differentiation reagents, such as retinoic acid (RA) [36], [37]. As seen from this, EC cell-derived pluripotent stem cells still carried the tumorigenic potential and they could escape apoptosis induced by the *Trp53* pathway [38].

The integration of specific signaling pathways into the definitions of the identities of pluripotent stem cells provided insights into the features of their transcriptional regulatory networks [39]. The core of the pluripotency network consisted of *Pou5f1*, *Sox2* and *Nanog*, while the LIF-STAT3 and BMP4-ID pathways could interact with the core members and the final balanced state among these genes maintained the self-renewal potential of the pluripotent stem cells [39], [40], [41], [42]. Because the expression levels of *Pou5f1* and *Sox2* in the EC cells were comparable to those of the ES cells, we shifted to study the Nanog, LIF-STAT3 and BMP4-ID pathways. Intriguingly, we found that *Nanog* and *BMP4* were both within the differentially expressed gene set, but not *Stat3* or *id*. Therefore, the core of the pluripotency network was better-maintained in the P19 cell-derived pluripotent stem cells, but the external signaling molecules, such as *BMP4,* were still somewhat aberrant. Nevertheless, P19 NTES cells underwent the pluripotency acquisition process, with *Nanog* as the typical indication [40]. *Nanog* has recently been found as the gateway keeper for the ground-state pluripotency and it played an essential role in the pluripotency acquisition during the generation of iPS cells and the conversion of epiblast-derived stem cells (EpiSCs) into ES cells [43]. Moreover, our results indicate that the induction of *Nanog* in P19 NTES cells was promoter region methylation-independent, which was different from its conventional regulatory mechanism. In contrast to *Nanog*, *H19* was induced in a DMR methylation-dependent pattern. *H19*, a paternally imprinted gene, played an important role in the developing embryo; and the dysfunction of *H19* always related to many diseases [44], [45]. In sum, these data provide strong evidence that diverse reprogramming mechanisms worked on different genes. The reprogramming of EC cells may be a complicated process and the experiences accumulated in this study may also provide clues for deciphering the reprogramming mechanisms.

There is increasing evidence that tumor tissues are hierarchically organized and sustained by a distinct subpopulation of cancer stem cells (CSCs). CSCs were first isolated from human acute myeloid leukemia [46], which provided an attractive model for investigating tumorigenesis. Similar paradigms suggested in solid tumors were further verified by isolating CSCs from these transformed tissues [47]. Different CSCs had specific growing environments, also called “niches”, and the properties of CSCs appeared to be influenced by their specific genetic mutations in a given tumor tissue as well as the progression of disease [48]. Therefore, the most challenging problem facing this field is the variability of CSCs coming from their origins. A possible solution to this challenge would be the identification of an *in vitro* model of CSCs. In this study, pluripotent cells were derived from cancer cells and we named them according to their origins, not their identities. In order to know their real identities, high-throughput method was used to find the differences among cancer cells, cancer cell-derived pluripotent cells and normal ES cells. The final results demonstrated that pluripotent cells derived from cancer cells were intermediates between cancer cells and ES cells. Although these intermediates were not fully pluripotent, they still could maintain the self-renewal ability *in vitro* and had distinct molecular characteristics. We predict that when these counterparts of cancer cells are used in drug screening, they might provide a new path for obtaining information about killing cancer stem cells.

At the same time, two additional complicated questions remain to be resolved: What is the difference between pluripotent cells from cancer cells and CSCs, and what is the hierarchy between them? It is our hope that our work will pave the way to obtain the counterpart of CSCs *in vitro* and perhaps it may help us understand the occurrence of tumors and the clinical treatment of cancers more thoroughly.

## Materials and Methods

### NT and NTES Cell Derivation

Specific pathogen free (SPF) grade mice were housed in the animal facility of the National Institute of Biological Sciences. Animal care and handling were conducted in accordance with policies promulgated by the Ethics Committee of the National Institute of Biological Sciences.

Metaphase II oocytes were collected from 8- to 10-week-old female B6D2F1 (C57BL/6 × DBA/2 F1) mice superovulated by administration of PMSG and hCG. Enucleation and injection were carried out with a piezo-driven micromanipulator system on an Olympus X 71 inverted microscopes. Reconstructed oocytes were activated in 10 mM Sr^2+^ in Ca^2+^-free medium containing 5 µg/ml cytochalasin B (Sigma, http://www.sigmaaldrich.com/), and cloned embryos were cultured in KSOM medium (Chemicon, http://www.chemicon.com/). The establishment of ES cells was done as previously described [49].

### Cell Culture

N2a and P19 cells (ATCC, http://www.atcc.org/) were cultured in ES medium consisting of DMEM (Chemicon) supplemented with 15% FBS (Hyclone, http://www.hyclone.com/), nonessential amino acids (Chemicon), 2-mercaptoethanol (Chemicon), and 1000 units/ml leukemia inhibitory factor (LIF) (Chemicon). P19 NTES cells and other kind pluripotent stem cells were all cultured in ES medium with mouse embryonic fibroblasts treated with mitomycin C (Sigma) pre-plated on the culture dishes.

### Karyotype Analysis

Cells were cultured in ES culture medium containing 0.05 µg/ml demecolcine (Sigma) for 5 h to arrest cells at M-phase. Then cells were trypsinized, pelleted by centrifugation and resuspended in hypotonic solution containing 0.4% NaCl and 0.4% citrate for 5 min. Freshly prepared methanol:acetic acid (3∶1) were used to fix the cells at room temperature for 40 min. Finally, the resuspended cells were placed onto pre-cleaned slides for chromosomes counting.

### RT-PCR

RT-PCR was performed to investigate the expression of pluripotency related genes. Trizol (Invitrogen, http://www.invitrogen.com/) was used to extract total RNA following the manufacturer's instructions. Then total RNA was treated with DNase before reversely transcribing to cDNA using the MLV reverse transcriptase system (Promega, http://www.promega.com.cn/). Primer sets for *Nanog*, *Pou5f1, Sox2* and *Zfp42* were designed as previously described [49].

### Western Blot

Protein samples were loaded onto 12% SDS-PAGE gel, resolved and electroblotted onto PVDF membrane. The membrane was blocked in Tris-buffered saline containing 0.1% Tween 20 and 5% low fat dry milk for 1 hour at room temperature. Then, the PVDF membrane was incubated with diluted primary antibodies at 4°C overnight. The peroxidase based detection was performed with Super Signal West Pico Chemiluminescent Substrate Kits (Pierce, http://www.piercenet.com/). Actin was used as the inner control.

Rabbit polyclonal anti-Nanog antibody was purchased from Upstate (Upstate, http://www.chemicon.com/). Rabbit polyclonal anti-Actin antibody was obtained from Sigma (Sigma).

### Teratoma Formation and Tetraploid Blastocyst Complementation

Teratoma formation was performed to evaluate the *in vivo* differentiation capacity of NTES cells. Cells distributed in 200 µl PBS (Invitrogen) were subcutaneously injected into the forelimb of SCID-beige mice. Approximately 4 weeks after injection, teratomas were examined histologically using standard protocols. Briefly, teratomas were dissected, weighed and fixed in 4% formaldehyde. The fixed samples were then embedded in paraffin and tissue sections were stained with hematoxylin and eosin for analysis (HE).

For the tetraploid aggregated assays, tetraploid blastocysts were first produced by electrofusion of 2-cell stage embryos of B6D2F1 mice and cultured in KSOM until blastocyst stage [50]. And ES cells or P19 cells were injected into tetraploid blastocysts using a piezo-actuated microinjection pipette. Then the tetraploid aggregated embryos were transfer back to KSOM medium for several hours and transplanted into the uterus of pseudo-pregnant mice.

### Real-time PCR

Briefly, Trizol (Invitrogen) was used to extract total RNA. All the manipulations were according to the manufacturer's instructions. Total RNA was treated with DNase (Chemicon) for 30 min, and then an equal amount of RNA was reverse transcribed to cDNA using the MLV reverse transcriptase (Promega). *Gapdh* was used as the internal control. SYBR green PCR master mix (TaKaRa, http://www.takara-bio.com/) was employed as the detector of fluorescence signal, and ABI 7500 was used in accordance to the manufacturer's instructions. One independent experiment contained three replicates of both targeted genes and inner control. The results of three independent experiments in duplicate were averaged to get the mean value of every gene. Paired Student's *t*-tests were performed to assess the statistical difference. The significant standard was set as: fold change >2; p-value <0.05. The primer pairs for real-time PCR were summarized in Table S1.

### Bisulfite Genomic Sequencing Analysis of DNA Methylation

TIANamp Micro DNA Kit (Tiangen, http://www.tiangen.com/) was used to extract DNA from 1000 tumor cells or ES cells; and the extracted DNA was treated by EpiTect Bisulfite Kit (QIAGEN, http://www.qiagen.com/) according to the manufacturer's instructions. Bisulfite modification treatments were triplicated for each sample. Then the treated samples were used as the template of nest PCR by using Takara Tag HS (TaKaRa). Primer sets for nest PCR were showed in Table S1. PCR products were recovered using Wizard SV Gel and PCR Clean-Up System (Promega). Then the PCR products were cloned into pMD18-T vector (TaKaRa) and sequenced by using ABI PRISM 3100 Genetic Analyzer (Applied BioSystems, http://www.appliedbiosystems.com/).

### Methylation Sensitive Restrictive Enzyme Digestion Assay of DNA Methylation

Methylation sensitive restrictive enzyme digestion was performed to analyze the methylation profiles of *Pou5f1*. TaqI (New England Biolabs, http://www.neb.com/) was used in this assay to recognize the two sites at the promoter region of *Pou5f1*. The digested fragments were electrophoresed on 2.5% agarose gels containing ethidium bromide.

### Microarray Data Processing and Statistical Analysis

In brief, we collected P19 cells and feeder free ES cells from 10 cm diameter culture dish. The collected cells were used for RNA extraction in Trizol (Invitrogen) reagent. Per sample contained 3 replicates. GeneChip® Gene 1.0 ST Array System (http://www.affymetrix.com) was used for microarray assays. For the data processing and analysis, R/BioConductor was first performed to analyze the raw data of microarray, and Robust Multichip Average (RMA) was used for the normalization (scale median = 500). Then, 1-way ANOVA (MATLAB 7.5) (MathWorks, Inc. http://www.mathworks.com/) was used to identity the differentially expressed genes. The significance standard was set as: fold change >2, p-value <0.01. PCA was also performed to evaluate the repeatability of every sample.

For the hierarchical clustering analysis of significantly changed genes, raw signal values were log2-transformed, centered relative to the median, with the parameters that the distance was a Euclidean distance and the linkage was the average.

Expression Analysis Systematic Explorer (EASE) was used to analyze gene ontology and KEGG pathways. Over representation of genes in a gene ontology node or a KEGG pathway is present if a larger fraction of genes within that gene ontology node/pathway is differentially expressed compared with all genes. An EASE score ≤0.05 was set as the cutoff [51].

## Supporting Information

Table S1 Primer sets for Real-time PCR and nest PCR.
(0.05 MB DOC)

Figure S1 Differentially expressed genes of GeneSet B in P53 signaling pathway. Genes in GeneSet B were mapped to the KEGG signaling pathway database; and the overrepresented genes were marked in yellow, with the non-corresponding ones marked in green. p-value < 0.05.
(1.07 MB TIF)

Figure S2 Differentially expressed genes of GeneSet C in P53 signaling pathway. Genes in GeneSet C were mapped to the KEGG signaling pathway database; and the overrepresented genes were marked in blue, with the non-corresponding ones marked in green. p-value < 0.05.
(1.07 MB TIF)

Dataset S1 Normalized Microarray Expression Data for GeneSet A.
(0.87 MB XLS)

Dataset S2 Raw data of Real-time PCR for the Verification of Microarray results.
(0.03 MB XLS)

Dataset S3 Normalized Microarray Expression Data for GeneSet B.
(0.18 MB XLS)

Dataset S4 Normalized Microarray Expression Data for GeneSet C.
(0.38 MB XLS)

Dataset S5 Overlapping Analysis Data of GeneSetA, B, C.
(0.11 MB XLS)

Dataset S6 Gene Ontology Analysis of Molecular Function.
(0.03 MB XLS)

Dataset S7 Gene Ontology Analysis of Biological Process.
(0.03 MB XLS)

Dataset S8 Gene Ontology Analysis of Cellular Component.
(0.03 MB XLS)

Dataset S9 Pathways Analysis Results of GeneSet A.
(0.02 MB XLS)

Dataset S10 Pathways Analysis Results of GeneSet B.
(0.01 MB XLS)

Dataset S11 Pathways Analysis Results of GeneSet C.
(0.01 MB XLS)
