# Supplementary material for: Linking Incomplete Reprogramming to the Improved Pluripotency of Murine Embryonal Carcinoma Cell-Derived Pluripotent Stem Cells
Source: PLoS One. 2010 Apr 26;5(4):e10320. doi: 10.1371/journal.pone.0010320 (PMC2859941; doi:10.1371/journal.pone.0010320)
Supplement: Table S1 — Primer sets for Real-time PCR and nest PCR. (0.05 MB DOC) [file pone.0010320.s001.doc]

**Table S1**. Primers sets for real-time PCR and nest PCR

| **Gene** | **Gene ID** | **Primer type** | **Primer sequence** |
| --- | --- | --- | --- |
| *H19* | NR_001592 | Real-time PCR | F: GTATGCCCTAACCGCTCAGTC;  R: CCAACCTCCCTCCCTAGAAAC |
| *Peg3* | NM_008817 | Real-time PCR | F: TCAATGACCTCACAAGCCACCAG;  R: CGGGCAACAGAGCGATGAAAGC |
| *Pou5f1* | NM_013633 | Real-time PCR | F: AATGCCGTGAAGTTGGAGAAGG;  R: AAAGAGAACGCCCAGGGTGAGC |
| *Nanog* | NM_028016 | Real-time PCR | F: GATGCGGACTGTGTTCTCTC;  R: GCTTGCACTTCATCCTTTGG |
| *Klf4* | NM_010637 | Real-time PCR | F: CCAAAGAGGGGAAGAAGGTCG;  R: GTGCCTGGTCAGTTCATCGG |
| *Sox2* | NM_011443 | Real-time PCR | F: CAGCATGATGCAGGAGCAGC;  R: CTGGAGTGGGAGGAAGAGGT |
| *c-Myc* | NM_010849 | Real-time PCR | F: GACTGTATGTGGAGCGGTTTC;  R: GCTGTCGTTGAGCGGGTAG |
| Trp53 | NM_011640 | Real-time PCR | F: GCTTTGAGGTTCGTGTTTGTG;  R: CTGGAGTGAGCCCTGCTGTC |
| *Bmp4* | NM_007554 | Real-time PCR | F: CGGTGGGAAACTTTCGATGTG;  R: CTTCTGCGGGTCAAGGTATGG |
| *Fbxo15* | NM_015798 | Real-time PCR | F: CTTTCTTACTTGGATGCGGTGAC;  R: GACTGAGGCTGTTGGTGAGGG |
| Gapdh | NM_008084 | Real-time PCR | F: TCTTGGGCTACACTGAGGAC;  R: CATACCAGGAAATGAGCTTGA |
| *Dppa5a* | NM_025274 | Real-time PCR | F: AGGGATCTCGAATGTCTCA;  R: TAGCCCGAATCTTGTTGTT |
| *Esrrb* | NM_011934 | Real-time PCR | F: TGGACTCGCCGCCTATGTT;  R: TCGATGTTGCCTTGAATGG |
| *H19* |  | Methylation | F1: GAGTATTTAGGAGGTATAAGAATT;  R1: ATCAAAAACTAACATAAACCCCT  F2: GTAAGGAGATTATGTTTATTTTTGG;  R2: CCTCATTAATCCCATAACTAT |
| *Peg3* |  | Methylation | F1: TGATAATAGTAGTTTGATTGGTAGGG;  R1: TAATTCACACCTAAAACCCTAAAACC  F2: TTTTGTAGAGGATTTTGATAAGGAGG;  R2: AAATACCACTTTAAATCCCTATCACC |
| *Pou5f1* |  | Methylation | F1: GAGGATTGGAGGTGTAATGGTTGTT;  R1: CTACTAACCCATCACCCCCACCTA  F2: TGGGTTGAAATATTGGGTTTATTT;  R2: CTAAAACCAAATATCCAACCATA |
| *Nanog* |  | Methylation | F: AAGTATGGATTAATTTATTAAGGTAGTT;  R1: AAAAAACCCACACTCATATCAATATA;  R2: CAACCAAATAACCTATCTAAAAA |
